# Changes in Energy Metabolism Trigger Pupal Diapause Transition of *Bactrocera minax* After 20-Hydroxyecdysone Application

**DOI:** 10.3389/fphys.2019.01288

**Published:** 2019-10-30

**Authors:** Yong-Cheng Dong, Zhen-Zhong Chen, Anthony R. Clarke, Chang-Ying Niu

**Affiliations:** ^1^Key Laboratory of Biology and Sustainable Management of Plant Diseases and Pests of Anhui Higher Education Institutes, College of Plant Protection, Anhui Agricultural University, Hefei, China; ^2^Hubei Insect Resources Utilization and Sustainable Pest Management Key Laboratory, College of Plant Science and Technology, Huazhong Agricultural University, Wuhan, China; ^3^Faculty of Science and Technology, School of Earth, Environmental and Biological Sciences, Queensland University of Technology, Brisbane, QLD, Australia

**Keywords:** comparative proteomics, diapause termination/transition, 20-hydroxyecdysone, dormancy responses, seasonal adaptation

## Abstract

Correct timing of diapause entry and exit is critical for a species' survival. While many aspects of insect diapause are well-studied, the mechanisms underlying diapause termination remain largely unknown. The Chinese citrus fly, *Bactrocera minax*, is a univoltine insect with an obligatory pupal diapause. The application of 20-hydroxyecdysone (20E) is known to terminate diapause in *B. minax*, and we used this approach, along with isobaric tags for relative and absolute quantitation technology, to determine the proteins associated with diapause termination in this fly. Among 2,258 identified proteins, 1,169 proteins significantly differed at 1, 2, and 5 days post-injection of 20E, compared with the solvent-injected control group. Functional annotation revealed that the majority of differentially expressed proteins were enriched in the core energy metabolism of amino acids, proteins, lipids, and carbohydrates as well as in signal transduction pathways including PPAR signaling, Calcium signaling, Glucagon signaling, VEGF signaling, Ras signaling, cGMP-PKG signaling, and cAMP signaling. A combined transcriptomic and proteomic analysis suggested the involvement of energy metabolism in the response of diapause transition. RNA interference experiments disclosed that a 20E injection triggers diapause termination probably through non-genomic actions, rather than nuclear receptor mediated genomic actions. Our results provide extensive proteomic resources for insect diapause transition and offer a potential for pest control by incapacitating the regulation of diapause termination either by breaking diapause prematurely or by delaying diapause termination to render diapausing individuals at a high risk of mortality.

## Introduction

Diapause is a state of developmental arrest evolved by organisms to survive predictable inclement conditions (notably, but not-exclusively, winter) and widely occurs in insects and other invertebrates (Tauber and Tauber, 1976; MacRae, 2010). Insect diapause is pre-programmed in response to environmental or genetic factors and affords the diapausing organisms the ability not only to mitigate the seasonal stresses of hostile periods but also to synchronize their life cycle with the availability of key resources (Denlinger, 2000). While successful entry and exit from diapause is critical to individual survival, subtle phenotypic, and genotypic variation in the timing of diapause can have significant population level impacts. For example, in the *Rhagoletis pomonella* sympatric speciation model, population divergence from the ancestral host plant hawthorn to the new host, apple, is thought to have been facilitated by changes in pupal diapause emergence, which better synchronizes the divergent apple fly populations with apple fruiting (Dambroski and Feder, 2007; Feder et al., 2010).

However, insects display divergent phenotypic plasticity of diapause among species. On the basis of the decision to enter diapause, insect diapause is traditionally categorized into two types: facultative and obligatory diapause (Tauber and Tauber, 1976; Denlinger, 2002). In facultative diapausing species, the diapause switch (entry or exit) is elicited by the perception of environmental cues, commonly photoperiod and temperature, which are subsequently translated into endocrine signals: hence the concept of neurohormonally regulated diapause ontogenesis (Hodek, 1996; Denlinger, 2002; Denlinger et al., 2005). However, in the second diapause type, obligatory diapause, diapause is a fixed component of the lifecycle where the “decision” of diapause entry or exit is hardwired, requiring no external diapause-influencing cues (Denlinger et al., 2005; Koštál et al., 2016).

For obligate diapausing species, evidence confirmed the variable developmental patterns within a species (Dambroski and Feder, 2007; Ragland et al., 2009; Dong et al., 2013), yet the molecular and physiological events of diapause regulation that translate the decision of diapause enter/exit are poorly understood (Denlinger, 2002; Denlinger et al., 2005; Meyers et al., 2016; Koštál et al., 2017). In this paper we focus on the switch(s) associated with diapause exit. Two main hypotheses have been proposed to explain diapause termination in obligate diapausers. Firstly, the pupal energy reserve hypothesis (Feder et al., 2010) suggests that diapausing individuals lower their metabolic rate to minimize energy needs but monitor their energetic reserves to ensure that there remains a critical energy reserve level to allow fir successful metamorphosis during the post-diapause period; however, the intricate physiological processes associated with energy sensing are poorly characterized. The second major hypothesis promotes the presence of a cold-degree-day counting mechanism (Hodek and Hodková, 1988; Hodek, 2002). A certain number of cold degree days experienced allows the diapausing individual to bridge the refractory period of obligate diapause, after which they become more sensitive to environmental cues, and the nervous or endocrine systems then take over the regulation of diapause terminating events as for a facultative diapauser.

*Bactrocera minax* (Enderlein) (Diptera: Tephritidae) is a univoltine fruit fly species located in the eastern Himalayan region and adjoining temperate zones to the north and south. The fly has an obligatory pupal diapause for overwintering, and the spring emergence of adults is closely linked to the phenology of a restricted number of *citrus* species, which are the only known hosts (Dorji et al., 2006; Zhou et al., 2012; Dong et al., 2014b). A mandatory chilling experience is required for diapausing *B. minax* individuals to break diapause before moving pupae into permissive conditions (Dong et al., 2013). However, artificial application of 20-hydroxyecdysone (20E) can eliminate the species' obligate diapause quickly (Dong et al., 2013; Chen et al., 2016). This is similar to the case of developmental arrest in diapausing pupae of the flesh fly, which is caused by a halt of ecdysteriod production and can be rescued by the application of ecdysteriods (Denliger et al., 1980).

Hormonal manipulation that triggers the rapid response of diapause termination provides the opportunity to identify the early molecular events of diapause transition. The variation of 20E sensitivity along the course of the diapause of *B. minax* was negligible since the sigmoid adult emergence curves of groups treated with 20E at different months were nearly overlapped (Chen et al., 2016), implying a rapid response involved in its diapause termination. However, considering the different physiological modulations mediated by steroid hormones (Lösel and Wehling, 2003; Spindler et al., 2009), here we pose a question as to whether exogenous 20E application in *B. minax* (1) merely offsets the shortage of ecdysone in diapausing individuals and binds to the heterodimeric ecdysone receptor complex Ecr/Usp to initiate adult morphogenesis, or (2) forwards a signal to directly modulate endocrine activity or metabolism to resume development via other actions rather than the classic nuclear receptor complex Ecr/Usp. Although artificial 20E manipulation is very successful in terminating diapause in *B. minax*, as it is for other obligatory diapausing insects (Kidokoro et al., 2006), the mechanisms underlying the invoked diapause transition remain poorly understood.

During the past decade, omic approaches, including transcriptomics (Poelchau et al., 2013; Hao et al., 2017; Koštál et al., 2017), proteomics (Lu and Xu, 2010; Tu et al., 2015; Tan et al., 2017; Zhao et al., 2017), and metabolomics (Zhang et al., 2012; Wang et al., 2017), have all been applied or integrated to rapidly expand our understanding of the mechanisms of diapause regulation by comparing non-diapausing individuals with diapausing counterparts. These studies mainly focused on the diapause induction and preparation phases, revealing that the stress tolerance related and metabolic pathways were selectively expressed. But there are only a few attempts to explore the candidates of genes or pathways at a transcriptional level for the regulation of diapause termination (Ragland et al., 2010, 2011). Similarly to diapause induction, termination can be directly stimulated or pre-programmed by environmental cues (Ragland et al., 2011). As it was thought an excellent approach to identify downstream gene expression rather than processes that occur upstream of ecdysteroid pulse of diapause termination, the complex molecular events and interactions of pharmacologically manipulated diapause termination remain largely unknown.

Proteins, as the most versatile functional macromolecules, are involved in virtually all biological processes in living cells and are the focus of this study. While mRNA levels code for proteins, and proteins can thus be studied indirectly using transcriptomes, studies in non-model systems have reported low correlation between proteins and mRNA levels (Haider and Pal, 2013; Tu et al., 2015; Zhao et al., 2017; Ziv et al., 2017). We therefore used the isobaric tags for relative and absolute quantitation (iTRAQ) methods to quantify the changes of both common and less abundant proteins across experimental treatments. Specifically, we assessed protein changes in *B. minax* pupae in a time series, i.e., 1, 2, and 5 days after 20E application, as it was reported 24 h may not have been sufficient to manifest a full transcriptional response to the hormone (Ragland et al., 2010). Our hypothesis was that 20E manipulation would elicit pleiotropic pharmacological effects to terminate diapause in *B. minax*, which may go beyond the prevailing model of diapause caused by a simple cease in the release of single hormone. Therefore, the current study aims to quantify the changes of proteins at different time points after 20E application, expecting to pinpoint molecular regulation and signaling cascades with rapid diapause termination. Given that there is limited phylogenetic conservation of diapause across species (Ragland and Keep, 2017), deciphering the underlying mechanisms of diapause regulation in *B. minax* would consequently provide an important basis for research related to stress adaptation and shed new light for the applications of pest control against this and other species (Denlinger, 2008).

## Materials and Methods

### Preparation of Materials

#### Insect Collection and 20-hydroxyecdysone Microinjection

Fruits infested with *B. minax* maggots were collected from an abandoned citrus orchard in Sandouping (Latitude: 30.81, Longitude: 111.05) and placed in a field to recover pupae, as with our previous study (Dong et al., 2013). Pupal diapause can be rapidly terminated by 20E topical application or microinjection (Dong et al., 2014a; Chen et al., 2016), microinjection has a more effective function on diapause termination. Newly formed pupae (<2 weeks) were artificially manipulated to terminate diapause via a 20E injection. 20-hydroxyecdysone was dissolved in 100% ethanol and then diluted to 1 μg/μl in 10% alcohol for the following injection. Pupae were first surface sterilized by 1% bleach and washed by sterilized water. After drying, each pupa was penetrated by a fine metal microinjection needle and then syringed with 0.2 μl 20E solution (World Precision Instruments, FL). The treated pupae were placed in an incubator with a temperature of 22 ± 1°C and a photoperiod of 14L:10D. After 20E treatment, individuals at different time-points including 1, 2, and 5 days post-injection (DPI) were sampled, and individuals treated with 0.2 μl carrier solution (10% alcohol) serve as a control group. Ten individuals at each time-point were pooled for protein extraction and each time-point was replicated twice.

### Protein Analyses

#### Protein Extraction

After removal of the puparium, the whole body of the pupa was disrupted in a lysis buffer (8 M urea, 40 mM Tris-HCl with 1 mM phenylmethane sufonyl fluoride, 2 mM EDTA and 10 mM dithiothreitol, pH 8.5) with a protease inhibitor by TissueLyser (Huang et al., 2018). After centrifuging at 25,000 g for 20 min, the supernatant was carefully removed and mixed with 5 vol of cold acetone and stored at −20°C overnight. The mixture was centrifuged at 4°C 15,000 g for 20 min. The pellets were dissolved with a lysis buffer, 1 mM PMSF, 2 mM EDTA, and 10 mM DTT and then ice-cooled for 5 min. After being centrifuged at 4°C 25,000 g for 20 min, the supernatant was mixed with DTT at a final concentration of 10 mM and kept at 56°C for 1 h to reduce the disulphide bond of peptides. Subsequently, 55 mM IAM was added and kept in darkness for 45 min. Five-fold volumes of chilled acetone was added and maintained at −20°C for 2 h. After centrifuge at 4°C 25,000 g for 15 min the protein pellet was dissolved with a lysis buffer. Protein concentration and integrity was checked using the Bradford method and SDS-PAGE analysis prior to quantitative proteomic analysis (Supplementary Image S1).

#### Protein Digestion and Sample Labeling

Firstly, 100 μg protein was digested by Trypsin Gold with the ratio of protein/trypsin (20:1) at 37°C for 4 h; we then added Trypsin Gold again and it digested for 8 h. After trypsin digestion, the peptides were centrifuged and re-dissolved with 0.5 M TEAB for iTRAQ (Isobaric tags for relative and absolute quantitation) labeling. Sample labeling with isobaric tag was carried out according to the manufacturer's protocol (iTRAQ® Reagents−8plex kit, Applied Biosystems/MDS Sciex, Foster City, CA). The peptide samples were labeled with respective isobaric tags and incubated for 2 h and subjected to peptide fractionation. Samples were labeled as follows: iTRAQ 113/114, control (CK); iTRAQ 115/116, 1 DPI (1d); iTRAQ 117/118, 2 DPI (2d); iTRAQ 119/121, 5 DPI (5d).

#### Peptides Fractionation by Reverse Phase Chromatography

Briefly, using the Shimadzu LC-20AB HPLC system, the peptides were reconstituted with buffer A (5% CAN, 95% H_2_O, adjust pH to 9.8 with ammonia) to 2 ml and loaded onto a 4.6 × 250 mm Gemini C 18 column containing 5 μm particles (Phenomenex). The peptides were eluted at a flow rate of 1 ml/min with a gradient of 5% buffer B (5% H_2_O, 95% CAN, adjust pH to 9.8 with ammonia) for 10 min, 5–35% buffer B for 40 min, 35–95% buffer B for 1 min. The system is then maintained at 95% buffer B for 3 min and decreasing to 5% within 1 min before equilibrating with 5% buffer B for 10 min. Elution is monitored by measuring absorbance at 214 nm, and fractions are collected every 1 min. The eluted peptides are pooled as 20 fractions and vacuum-dried. Each fraction was re-suspended in a buffer of 5% CAN + 0.1% FA and centrifuged at 20,000 g for 10 min.

#### LC-ESI-MS/MS Analysis Based on Q EXACIVE

Each fraction was re-suspended in buffer A (5% CAN, 0.1% FA) and centrifuged at 20,000 g for 10 min. The final concentration of peptides was about 0.5 g/l. The supernatant was loaded on a LC-20AD nanoHPLC (Shimadzu, Kyoto, Japan) by the autosampler onto a 2 cm trap column. Then, the peptides were eluted onto an 18 cm analytical C18 column (inner diameter 75 m, packed in-house). The samples were loaded at 8 l/min for 4 min, continued by a 41 min gradient running at 0.3 μl/min from 5 to 35% buffer B (98% CAN, 0.1% FA), followed by 5 min linear gradient to 80% buffer B, maintained at 80% for 5 min, and finally returned to 5% within 1 min. The peptides were subjected to nanoelectrospray ionization followed by tandem mass spectrometry (MS/MS) in a Q EXACTIVE (Thermo Fisher Scientific, San Jose, CA) coupled online to the HPLC. Intact peptides were detected in the Orbitrap at a resolution of 70,000. Peptides were selected for MS/MS using the high-energy collision dissociation (HCD) operating mode with a normalized collision energy setting of 27.0; ion fragments were detected in the Orbitrap at a resolution of 17,500. A data-dependent procedure that alternated between one MS scan followed by 15 MS/MS scans was applied for the 15 most abundant precursor ions above a threshold ion count of 20,000 in the MS survey scan with a following Dynamic Exclusion duration of 15 s. The electrospray voltage applied was 1.6 kV. Automatic gain control (AGC) was used to optimize the spectra generated by the Orbitrap. The AGC target for full MS was 3e6 and 1e5 for MS2. For MS scans, the m/z scan range was 350–2,000 Da. For MS2 scans, the m/z scan range was 100–1,800.

#### Analysis of Spectra and Protein Quantification

The raw MS/MS data was converted into MGF format by the thermo scientific tool Proteome Discover and the exported MGF files were searched against our previously constructed transcriptome database for protein identification with trypsin specificity using the Mascot (v.2.3.02) search algorithm (Matrix Science Inc., Boston, MA; Brosch et al., 2009), allowing one missed cleavage and fixed modifications at lysine residues, carbamidomethyl, and N-termini of the peptides in the MS/MS Ion search. Mass tolerance was set to 20 ppm for precursor ions and 0.05 Da for fragment ions. An automated software, IQuant, which integrates Mascot Percolator and advanced statistical algorithms to process the MS/MS signals generated from the peptides labeled by isobaric tags, was applied for protein quantitation (Wen et al., 2014).

#### Differentially Expressed Proteins (DEPs) Screening and Functional Annotation

Differentially expressed proteins were filtered with >1.2-fold at *Q*-value (the corrected *P*-value) < 0.05. Final DEPs were determined in at least one replicate and the remaining replicate has consistent expression. Functional annotation of Gene Ontology (GO), Clusters of Orthologous Groups (COG), and Kyoto Encyclopedia of Genes and Genomes (KEGG) pathway enrichment were performed.

### Integrated Analyses of Proteomic and Transcriptomic Data

#### Correlation Analyses of Protein and mRNA Expression

To evaluate the correlation between previous transcriptomic data (samples from control and 5 days post-20E treatment, i.e., 5d vs. ck, GenBank Sequence Accession: SRR1238397; Dong et al., 2014a) and the proteomic data of corresponding samples, we designated the following parameters to sift the proteins and genes with differentially expressed signals. Specifically, proteins matched to unique peptides with changes >1-fold at *Q* < 0.05, and mRNAs with changes >2-fold at *Q* < 0.001 were selected. Proteins were determined as correlations as long as they were detected at the transcriptional level. Integrated analyses of GO and KEGG pathway enrichment with a *Q* < 0.05 were considered significant.

### RNA Interference

To test the potential pathway involved in 20E mediated diapause transition, the proportion of diapause termination was inspected by RNA interference assay. 20E responsive genes including ecdysone nuclear receptor *ecr* and transcription factor *broad* were obtained by retrieving previously constructed *B. minax* transcriptomic data (Dong et al., 2014a). dsRNA preparation was performed following the methods previously described (Dong et al., 2016; Wang et al., 2018). The DNA templates for dsRNA synthesis were amplified by PCR using specific primers containing the T7 RNA polymerase promoter at both ends (Supplementary Table S1), following the program: 95°C for 5 min, followed by 30 cycles of 95°C for 30 s, 58°C for 30 s, and 72°C for 2 min, and a final extension step of 72°C for 10 min. Approximately 1 μg of purified DNA template was applied to produce the target dsRNA using the Transcript Aid T7 High Yield Transcription Kit (Thermo). The quality and size of the dsRNA were determined by agarose gel electrophoresis and spectrophotometer (Thermo). Then, 1 μg of dsRNA in 0.2 μl together with 0.2 μl 20E (aforementioned working concentration) were microinjected into each diapausing individual. A dsRNA against enhanced green fluorescent protein (*egfp*) was used as control. Each treatment contains four replicates and 15 individuals for each replicate. The treated pupae were placed in an incubator with temperature of 22 ± 1°C and photoperiod of 14L:10D. Four treated pupae were snap frozen in liquid nitrogen and ground for RNA extraction at 1, 2, and 3 days post the injection. The RNA was extracted using the RNA Extraction Kit RNAiso Plus (TAKARA). Reverse transcription of the total RNA into cDNA was carried out using ReverAid First Strand cDNA Synthesis Kit (Fermentas, EU). The relative expression of target genes was detected by quantitative real-time polymerase chain reaction (qRT-PCR) using SYBR Premix EX Tag Kit (Takara, Dalian) with primers (Supplementary Table S1) following the manufacturer's protocol on a 7,300 real-time PCR system (Applied Biosystems, USA).

## Results

### Proteins Identified With Diapause Transition

**Figure 1:**
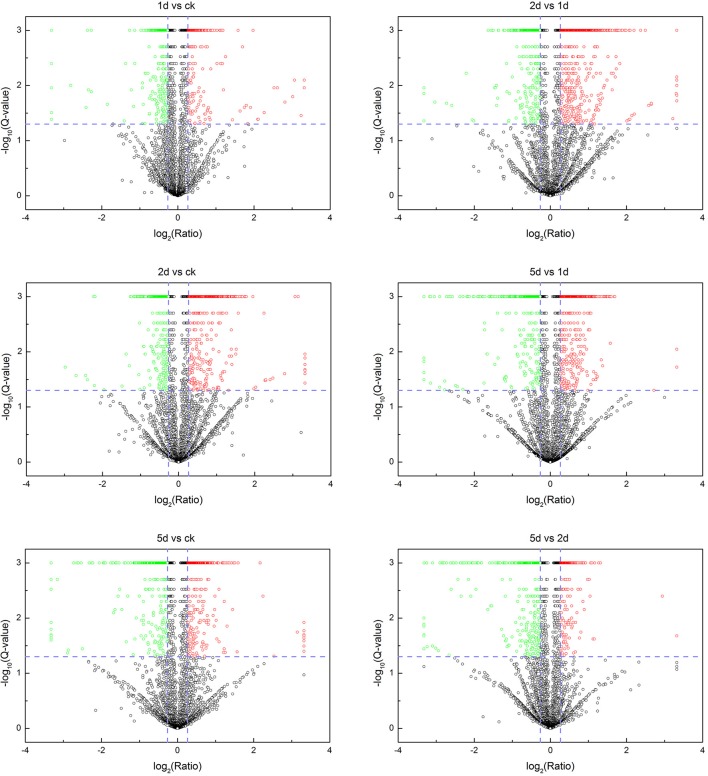
Volcano plots showing fold-change and *Q*-value for all identified proteins. Each panel shows a certain comparison and each dot represents a protein. Cutoff lines in blue indicate *Q* < 0.05 and fold change >1.2. Red scatters represent the significantly upregulated proteins, green scatters denote the significantly downregulated proteins, while black scatters indicate the non-significantly expressed proteins.

A total of 256,462 spectra were generated from eight time-resolved samples of tandem MS/MS assays, containing 57,983 unique spectra and 7,660 unique peptides (Table 1). In total, 2,258 proteins were identified through MS/MS ion search with a cutoff MascotPercolator *Q* ≤ 0.01 (Supplementary Table S2). Among them, 1,169 proteins (51.8% of total proteins) were found significantly up- or down-regulated in pairwise comparisons after 20E application with >1.2-fold change and *Q* < 0.05 (Figure 1, Table 2).

**Table 1:** Summary statistics of proteomic analysis in *Bactrocera minax*.

**Summary of statistics**	**Number**
Total spectra	256,462
Spectra	61,822
Unique spectra	57,983
Peptide	7,892
Unique peptide	7,660
Protein	2,258

**Table 2:** Changes in protein profile filtered with >1.2-fold change and *Q* < 0.05 in comparison to different days post-injection (DPI) of 20-hydroxyecdysone.

**Comparison**	**Up**	**Down**	**Total**
1d vs. CK	182	259	441
2d vs. CK	350	289	648
5d vs. CK	275	313	588
2d vs. 1d	442	307	749
5d vs. 1d	377	349	726
5d vs. 2d	165	288	453

*CK: control group treated with a carrier solution*.

**Figure 2:**
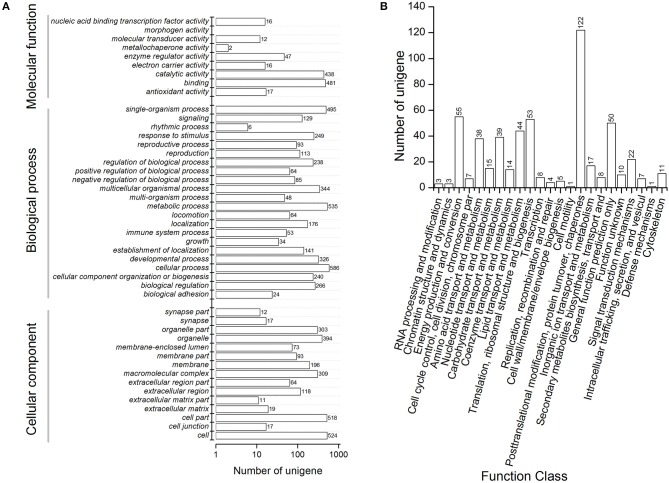
Functional annotation including **(A)** clusters of orthologous groups (COG) classification and **(B)** Gene Ontology (GO) terms of differentially expressed proteins in proteomic analysis of *Bactrocera minax*. Differentially expressed proteins were filtered with >1.2-fold change and *Q* < 0.05.

**Figure 3:**
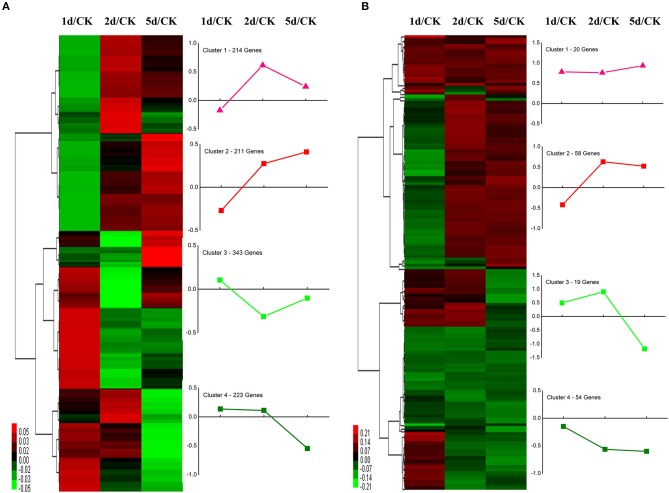
Clustering analysis of **(A)** the union and **(B)** the intersection of differentially expressed proteins (DEPs) at 1, 2, and 5 days post-injection (DPI) of 20-Hydroxyecdysone. Differentially expressed proteins were filtered with >1.2-fold change and *Q* < 0.05. The heatmaps show the changes of proteins over time compared with the control group. Each line represents one protein. Red color means upregulated and green color means downregulated. The line graph denotes the tendency of DEPs in each clustered branch.

We performed functional annotation of gene ontology (GO) and clusters of orthologous groups (COG) for these differentially expressed proteins. Among 1,169 DEPs, ~76.9 and 84.6% of the proteins were categorized into 46 functional GO groups and 23 COG categories, respectively (Figure 2, Supplementary Table S3). The top GO terms are “catalytic activity,” “binding,” “single-organism process,” “metabolic process,” “cellular process,” “cell,” and “cell part,” indicating these proteins exerted a dominant function of binding and catalytic activity in the metabolic process and the cellular process (Figure 2A). The largest COG group is “posttranslational modification, protein turnover, chaperones,” followed by “energy production and conversion,” “translation, ribosomal structure and biogenesis,” “general function prediction only,” “lipid transport and metabolism,” “carbohydrate transport and metabolism,” “amino acid transport and metabolism,” and “signal transduction mechanisms” (Figure 2B). Among all the identified DEPs, 991 proteins were found differentially expressed in the time course after 20E application, including 151 proteins, which were detected in all samples (Figure 3). Cluster analysis showed most of them were up-regulated, especially at 2-DPI of 20E injection. Several enzymes catalyzing the energy reactions, like maltase (CL2849.Contig2), phosphoglycerate kinase (Unigene15505), ATP synthase (Unigene36853) and pyruvate kinase (CL1646.Contig2), were switched on (see cluster 1 and 2 in Figure 3); while antimicrobial peptide e.g., Cecropin (Unigene15876, CL2782.Contig2), Attacin-B (Unigene14810, Unigene15032), Diptericin (CL718.Contig1), and heat shock proteins (Unigene11269, CL440.Contig4, Unigene15431, Unigene692) were down-regulated (see cluster three and four in Figure 3, more detail lists in Supplementary Table S4).

Kyoto Encyclopedia of Genes and Genomes (KEGG) pathway enrichment analysis showed that DEPs were involved in several biochemical pathways regarding protein turnover and carbohydrate and lipid metabolism post-20E treatment (Table 3, Supplementary Table S5). Apart from these energy metabolism-related pathways, signal transduction including Calcium signaling, Ras signaling and cGMP-PKG signaling *etc*. were notably enriched as well.

**Table 3:** Representative KEGG biochemical pathways enriched in comparisons through 1, 2, and 5 days post-injection (DPI) of 20-Hydroxyecdysone, compared with control group treated with a carrier solution.

**Comparison**	**KEGG gene set**
1d vs. CK	Metabolic pathways Glycerolipid metabolism Fructose and mannose metabolism PPAR signaling pathway* Amino sugar and nucleotide sugar metabolism Galactose metabolism Glyoxylate and dicarboxylate metabolism Calcium signaling pathway* Fatty acid metabolism cGMP-PKG signaling pathway* Tryptophan metabolism Fat digestion and absorption Pentose and glucuronate interconversions Protein processing in endoplasmic reticulum
2d vs. CK	Pentose phosphate pathway Glucagon signaling pathway* Pyruvate metabolism Ascorbate and aldarate metabolism Valine, leucine and isoleucine degradation Arginine and proline metabolism cGMP-PKG signaling pathway*
2d vs. 1d	Ribosome Pentose and glucuronate interconversions Riboflavin metabolism Tyrosine metabolism Serotonerigic synapse Fatty acid elongation Pentose phosphate pathway
5d vs. CK	Riboflavin metabolism Tyrosine metabolism Glycolysis/Gluconeogenesis Protein processing in endoplasmic reticulum Pentose phosphate pathway Glycerolipid metabolism
5d vs. 1d	Riboflavin metabolism Protein processing in endoplasmic reticulum VEGF signaling pathway* Pentose phosphate pathway Glycerolipid metabolism Fructose and mannose metabolism Pentose and glucuronate interconversions
5d vs. 2d	Ras signaling pathway* Glyoxylate and dicarboxylate metabolism cGMP-PKG signaling pathway* cAMP signaling pathway* Serotonergic synapse

*Only representative functional groups with Q value < 0.05 are listed. The asterisk indicates the signal transduction pathway*.

### Correlation Analyses of Proteins and mRNA Expression

**Figure 4:**
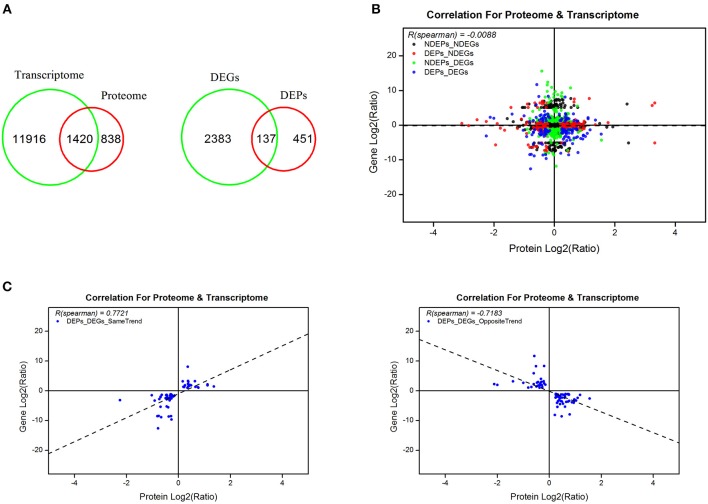
Combined analyses of proteomic and previously RNA-seq data at 5 days post-injection (DPI) of 20-hydroxyecdysone. **(A)** An overview of identified genes, proteins and differentially expressed genes and proteins in transcriptome and proteome; **(B)** correlation of the identified proteins and corresponding genes in proteomic and transcriptomic profiles; **(C)** correlation of the differentially expressed proteins with same and opposite trends of the corresponding transcripts. Scatter dots represent the proteins and the corresponding genes.

From the omics data of samples of 5 days post-20E treatment and the control group, we found 1,420 genes that correlated and 137 genes that differed significantly in response to 20E application (Figure 4A, Supplementary Table S6). There was no correlation between total proteins and mRNA levels (*r* = −0.0088, *df* = 1419, *P* = 0.74; Figure 4B). However, when the data was explored further, highly significant correlations were found. A total of 59 genes exhibited an expected expression pattern for proteins, *i.e*., up-regulated genes resulted in higher expression of protein, and the down-regulation of genes decreased protein expression (for these 59 genes, *r* = 0.7721, *df* = 58, *P* < 0.0001; Figure 4C, Supplementary Table S7). However, reverse patterns were also observed; 78 genes displayed the opposite expression pattern (for these 78 genes, *r* = −0.7183, *df* = 77, *P* < 0.0001; Figure 4C, Supplementary Table S8).

**Figure 5:**
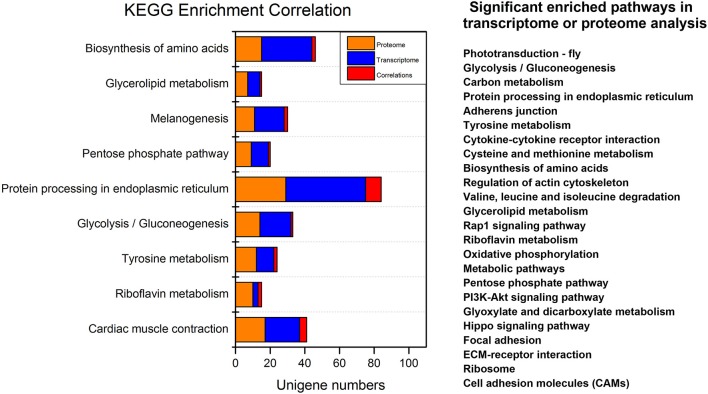
Overview of the significant enriched pathways of integrated transcriptomic and proteomic analyses. For simplicity we removed pathways related to human disease. Significant enriched pathways in only one omic analysis (transcriptome or proteome) are listed on the right.

The integrated analyses of GO and KEGG enrichment showed that the “structural molecular activity” in the molecular function category of GO classification and “Alzheimer's disease,” “Hypertrophic cardiomyopathy,” “Dilated cardiomyopathy,” “Legionellosis,” and “Cardiac muscle contraction” metabolic pathways were both significant in the transcriptome and the proteome (Supplementary Data Sheet 1). For simplicity we removed pathways related to human disease (Figure 5). Carbohydrate, protein and lipid metabolic pathways were found top enriched, suggesting the metabolic events were altered post-20E treatment for direct ontogenetic processes. Signaling transduction, including Rap1 signaling, PI3K signaling and Hippo signaling, was found significant in one of two omics analyses, and it was probably involved in the diapause terminating signals' transmission.

### RNAi Experiments

**Figure 6:**
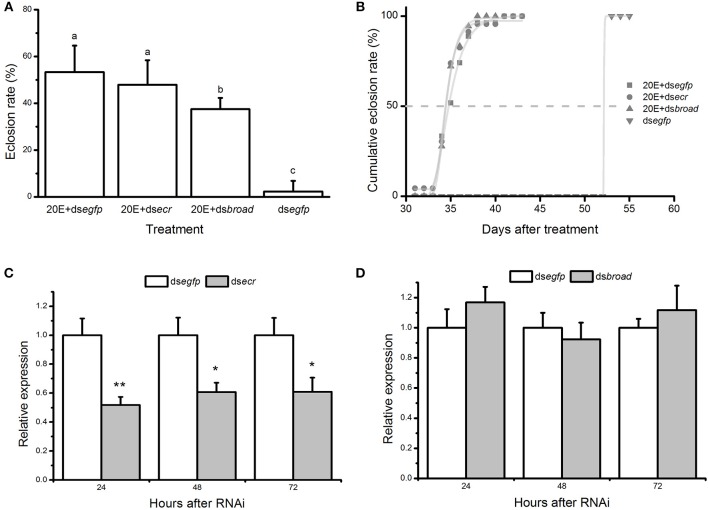
The effects of gene-silencing targeting to the 20E pathway on diapause termination. **(A)** The proportion and **(B)** accumulative emergence curve of *Bactrocera minax* adult eclosion under 22 ± 1°C within 60 days after injection of 20E + dsRNAs. Individuals injected with 20E + ds*egfp* and ds*egfp* serve as the controls. Mean values (± SD) were compared using one-way analysis of variance, and different letters represent the significant differences at *P* < 0.05 level; the transcriptional level of **(C)** *ecr* and **(D)** *broad* gene after injection of 20E and corresponding dsRNA. 20E + ds*egfp* serves as the control. Mean values (± SD) were compared using student *t*-test and the asterisk represents the significant difference at *P* < 0.05 level.

The results showed that an injection of 20E + dsRNA mixture can trigger an immediate response of diapause termination, which was reflected in a significantly elevated adult emergence within 60 days when compared to the control group [*F*_(3, 12)_ = 29.931, *P* < 0.001] and nearly overlapped sigmoid curves of adult emergence (Figure 6A). The direct ontogenetic processes of *B. minax* take 30–40 days if placed under ~24°C (Figure 6B). The expression of *ecr* but not *broad* was significantly reduced at 24, 48, and 72 h post-injection from qRT-PCR verification (Figures 6C,D), which did not affect the 20E-mediated diapause termination responses.

## Discussion

The adaptive timing of diapause is an evolutionarily robust strategy for insects, wherein the decision of diapause termination is a pivotal component to synchronize favorable conditions. Our time-resolved data demonstrated the diapause termination was closely associated with the energy metabolism of diapausing individuals, including core metabolic pathways of amino acids, proteins, lipids, and carbohydrate conversion. 20E-mediated diapause termination did not rely on the classic genomic action, probably through non-genomic action involving rapid changes of intracellular second messengers and signal transduction cascades.

The application of 20E allows the diapausing individuals to capacitate the direct development with significantly increased metabolism. Diapause is a state of metabolic depression with a remarkably lower respiration rate and elevated stress tolerance (Denlinger, 2000, 2002; King and MacRae, 2015). The elevated respiration rate is an apparent symbol characterizing the diapause termination of *B. minax* (Dong et al., 2014a; Wang et al., 2017), and is associated with resource allocation and morphogenesis (Hahn and Denlinger, 2007, 2011). Among 2,258 proteins identified, time-resolved data revealed that 1,169 proteins differed significantly across the series of time points after 20E application. It is not surprising that most DEPs were mainly enriched in core metabolic pathways including protein, sugar, and lipid metabolism (see Table 3). After 20E treatment, several enzymes and cytoskeleton proteins were upregulated while antibacterial peptides and heat shock proteins showed a declined pattern over time, reflecting the enhanced metabolism and reduced stress tolerance, a symbolic syndrome of diapause termination. Once the diapause-terminating signal was perceived, aerobic metabolism was enhanced and direct ontogenesis was activated immediately for resource rearrangement, preparing the ATP and precursor supply for cell growth and division (Figure 3B, Supplementary Table S4). Functional annotation showed that several enzymes were activated to boost catabolism, satisfying the energy and material requirement for accelerating ontogenetic processes (Figure 3, Supplementary Table S4). It was concordant with the typical syndrome of diapause termination, i.e., subsequent elevated metabolic respiration, implying that the diapause transition had begun.

Regarding the energy reserve hypothesis, one possibility regarding the involvement of enhanced metabolism in diapause termination would be that the incurred perturbation of nutrients, such as glucose content, may forward a signal to the brain to modulate the diapause processes. In other words, adjusted levels of metabolites in hemolymphs would be the main cause rather than the consequence of diapause termination in *B. minax*. For example, an artificially manipulated disorder of TCA (tricarboxylic acid cycle) intermediates and related metabolites: glucose and pyruvate, for example, can break the diapause in cotton bollworms (Xu et al., 2012). Pyruvate metabolism and the glucagon signaling pathway were significantly enriched at 2 days post-injection of 20E (Table 3). Insulin signaling is known to regulate carbohydrate and lipid metabolism (Saltiel and Kahn, 2001) and was thought to be the main “controller” in the energy-sensing of insect diapause (Hahn and Denlinger, 2011; Sim and Denlinger, 2013). The recent study showed that the major components of insulin signaling play important roles in diapause regulation (Denlinger et al., 2005; Sim and Denlinger, 2008, 2013; Sim et al., 2015) and maintaining glucose homeostasis in high or low levels is critical for diapause decision (Zhang et al., 2017). VEGF signaling was found significant at 5d vs. 1d comparison (Table 3). It participates in activating multiple downstream cascades, including the main branch of insulin signaling (that is, Ras-MAPK and PI3K-Akt cascades; Boucher et al., 2014). Similarly, adipokinetic hormones are believed to participate in nutrient homeostasis, particularly blood sugar regulation, and function in the diapause-associated changes in metabolism (Hahn and Denlinger, 2007). Lipids, as the dominant dormancy energetic storage (Hahn and Denlinger, 2011), were modulated after 20E treatment since some lipid metabolism pathways (including PPAR signaling), fat digestion and absorption, glycerolipid metabolism, fatty acid elongation, and fatty acid metabolism, were significantly assigned (Table 3). Diapausing *B. minax* are limited to relying on a pre-sequestered energy reserve as they are incapable of feeding and compensating for energy deficiency during diapause even when orchestrated diapause development proceeds optimally; thus the altered metabolism rate *per se* would be inferred to change the fate of diapausing individuals as a result of their direct effects on modified nutrients' levels.

Signaling transduction pathways account for the translating of diapause-terminating manipulation and transmitting the signals of diapause transition, which are of great importance in regulating the processes of diapause (Hao et al., 2016). As found in the previous studies (Dong et al., 2014a; Wang et al., 2014; Chen et al., 2016), 20E could trigger a rapid response of diapause termination, resulting in a higher synchronization of *B. minax* adult emergence, meaning it has an absolute advantage over the chilling treatment (Dong et al., 2013). 20E is a well-known ecdysteroid that initiates the insect molting process (Spindler et al., 2009), functioning by binding to a heterodimeric complex of nuclear receptors EcR/USP and activating the ecdysone regulatory cascade (Smagghe, 2009). But surprisingly the silence of ecdysone receptor *ecr* expression did not hamper the diapause-terminating effects of exogenous 20E application. Over time, the diapausing individuals (with gradually decreasing diapause intensity) become more sensitive to the application of 20E to the population, but responded similarly on an individual level (Chen et al., 2016). Thus, we suppose that the exogenous 20E did not simply replenish the lack of endogenous 20E to terminate the diapause. As a “late” 20E responsive gene, *broad* might be activated by both 20E genomic and non-genomic actions. Furthermore, *broad* is an ecdysone-induced transcription factor that was required for the pupal differentiation (Konopova and Jindra, 2008) and onset of metamorphosis in *Drosophila* (Zhou and Riddiford, 2002); it can regulate stem cells to generate adult cells during metamorphosis (Zeng and Hou, 2012) and was associated with diapause termination in *Chymomyza costata* (Koštál et al., 2017). Thus, the interruption of *broad* gene expression may have more severe impacts than *ecr* on the 20E signaling cascades, even though non-significant silencing effects were found in the present study. The reason for the insufficient silencing effect of the *broad* gene might be attributed to the amplification effect of the injected exogenous 20E; *broad* is an ecdysteriod response gene (Konopova and Jindra, 2008), and we found its transcriptional level was promoted after 20E manipulation (Chen et al., 2016). The interruption of *ecr* and *broad* expression failed to block or retard the diapause-terminating responses. It means knockdown of 20E signaling (the silencing of *ecr* or *broad*) did not substantially abate the transfer of the diapause terminating signals.

Steroid hormones are thought traditionally to modulate the transcription of mRNA and subsequent protein biosynthesis in a classic model of genomic actions (McEwen, 1992, 1994), while evidence for non-genomic effects has shown that steroids can operate independently or simultaneously with genomic actions (Wehling, 1997; Tomaschko, 1999; Lösel and Wehling, 2003; Norman et al., 2004; Smagghe, 2009). The response of steroids mediated by genomic actions, e.g., the modulation of gene expression, is known to take place from hours to even days, whereas non-genomic actions are much more rapid and occur in seconds or minutes (Schmidt et al., 2000; Lösel and Wehling, 2003). One possible explanation for the pattern of RNAi experiment is that the 20E-induced diapause transition was not reliant upon the genomic actions of steroid hormones, or not solely at least; non-genomic cell surface receptor-mediated signal transduction pathways were presumably involved.

We assume the involvement of 20E rapid non-genomic actions mediated the diapause termination, and there are no synergistic effects for the amounts of 20E to the initial action because the sigmoid curves with different treated dosages almost overlapped. In our previous study, 20E *per se* can elicit profound effects on diapause terminating regardless of the amount of 20E applied and the stages of diapausing pupae with diversified diapause intensity (Chen et al., 2016). As reported, non-genomic actions often alter the intracellular second messengers and signal transduction cascades (Lösel and Wehling, 2003). Signal transduction pathways, including Calcium signaling and Ras signaling, were modulated in response to 20E stimuli (e.g., CL1982.Contig2, Unigene15378, Unigene15912, CL1803.Contig2, Figure 3B, Supplementary Table S4), probably involving rapid changes of second messengers through non-genomic actions. It has also been found in other studies that steroid-generated Ca^2+^ signaling is functionally involved in diapause regulation (Hao et al., 2016; Zhao et al., 2017). Ras proteins translocate a number of signal molecules and activate multiple signaling transductions (Olson and Marais, 2000). The second messenger adenosine 3′, 5′ -monophosphate (cAMP) could induce short-term and long-term effects through different signal transduction cascades (Neves et al., 2002). These modulated second messengers may route the 20E signaling to distinct intracellular signaling to control diapause transition. In line with these findings, non-genomic and genomic actions may synergize, resulting in rapid onset and long-lasting persistence (Revelli et al., 1998). In addition, protein kinase pathways are also linked to non-genomic effects of steroids (Lösel and Wehling, 2003). 20E regulates the expression of Ca^2+^-dependent protein kinase and associated protein phosphorylation to promote insect metamorphosis (Chen et al., 2017). The changes in protein phosphorylation levels were considered to be a quick response to the metabolic depression of diapause (Lu and Xu, 2010).

From the perspective of thermoperiodism, cold chilling might be a driving force to evoke the production and accumulation of 20E in *B. minax*, resulting in a cline of diapause intensity (Masaki, 2002; Dong et al., 2013). Sufficient cold chilling can induce diapause termination in many insects (Hodek, 2002), like the process of vernalization in plants (Brunner et al., 2014). In plants, low temperature is favorable for 20E accumulation (Kayani et al., 2014). Calcium signaling is also known to insect cold sensing (Teets et al., 2013). 20E treatment and cold chilling may share some common components, e.g., calcium signaling to modulate diapause transition, although it is less efficient in cold exposure than pharmacological manipulation.

## Conclusions

In the present study, our quantitative proteome analysis provides a global view of proteins in response to pharmacological diapause-terminating treatment (20E injection) at various points in time. The results show the diapause transition in *B. minax* was associated with the energy metabolism (where most of DEPs were mainly assigned to energy metabolism and related regulation pathways) and signal transduction pathways. 20E-induced diapause termination did not rely on genomic action via an ecdysone nuclear receptor complex, but probably rather through membrane mediated non-genomic actions. Signal transduction pathways including Calcium signaling, Ras signaling and the cAMP signaling transduction pathway were presumably involved in diapause terminating signals' transmitting rapidly in response to 20E application. Our results provide extensive protein profiling for insect diapause termination and offer potentially important findings regarding pest control by incapacitating the regulation of diapause termination either by breaking diapause prematurely or by delaying diapause termination, ensuring that diapausing individuals are exposed to a high risk of mortality in this and other species.

## Data Availability Statement

The dataset and materials presented in the investigation is available by request from the corresponding author.

## Ethics Statement

Studies on invertebrates do not require ethical committee approval.

## Author Contributions

Y-CD and C-YN conceived and designed the study. Y-CD and Z-ZC performed the experiments and analyzed data. Y-CD, AC, and C-YN interpreted the results and wrote the manuscript. All authors read and approved the manuscript.

### Conflict of Interest

The authors declare that the research was conducted in the absence of any commercial or financial relationships that could be construed as a potential conflict of interest.

## Supplementary Material

Supplementary Table S1 The specific primers used for dsRNA preparation and qRT-PCR verification.

Supplementary Table S2 The abundance and function annotation of all identified proteins through MS/MS ion search with cutoff MascotPercolator *Q* ≤ 0.01.

Supplementary Table S3 The Gene Ontology (GO) and Clusters of Orthologous Groups (COG) functional annotation of differentially expressed proteins.

Supplementary Table S4 The intersection and union of differentially expressed proteins across different time points.

Supplementary Table S5 Kyoto Encyclopedia of Genes and Genomes (KEGG) pathway enrichment for all differentially expressed proteins.

Supplementary Table S6 The correlated genes in transcriptome and proteome data.

Supplementary Table S7 The significant expressed protein with same expression trend in transcriptome and proteome data.

Supplementary Table S8 The significant expressed protein with opposite expression trend in transcriptome and proteome data.

Supplementary Data Sheet 1 The GO and KEGG enrichment for the significant expressed proteins in transcriptome and proteome data.

Supplementary Image S1 SDS-PAGE electrophoresis of the samples of Bactrocera minax pupae treated by carrier solution and 20-hydroxyecdysone at different time points. The samples were separated by 10% resolving gel in duplicate.
