# Supplementary figures and images for: Changes in Energy Metabolism Trigger Pupal Diapause Transition of Bactrocera minax After 20-Hydroxyecdysone Application
Source: Front Physiol. 2019 Oct 30;10:1288. doi: 10.3389/fphys.2019.01288 (PMC6831740; doi:10.3389/fphys.2019.01288)

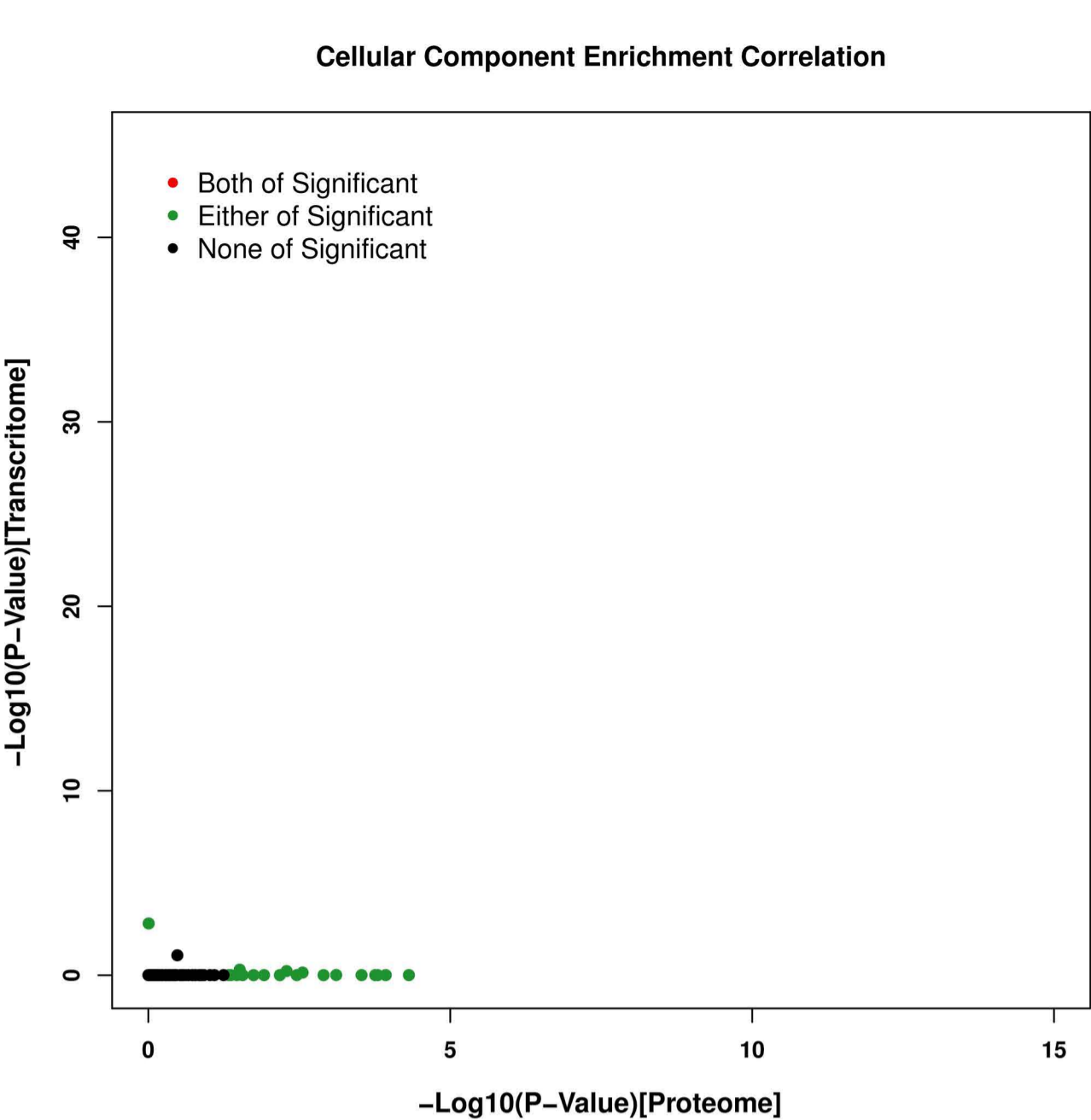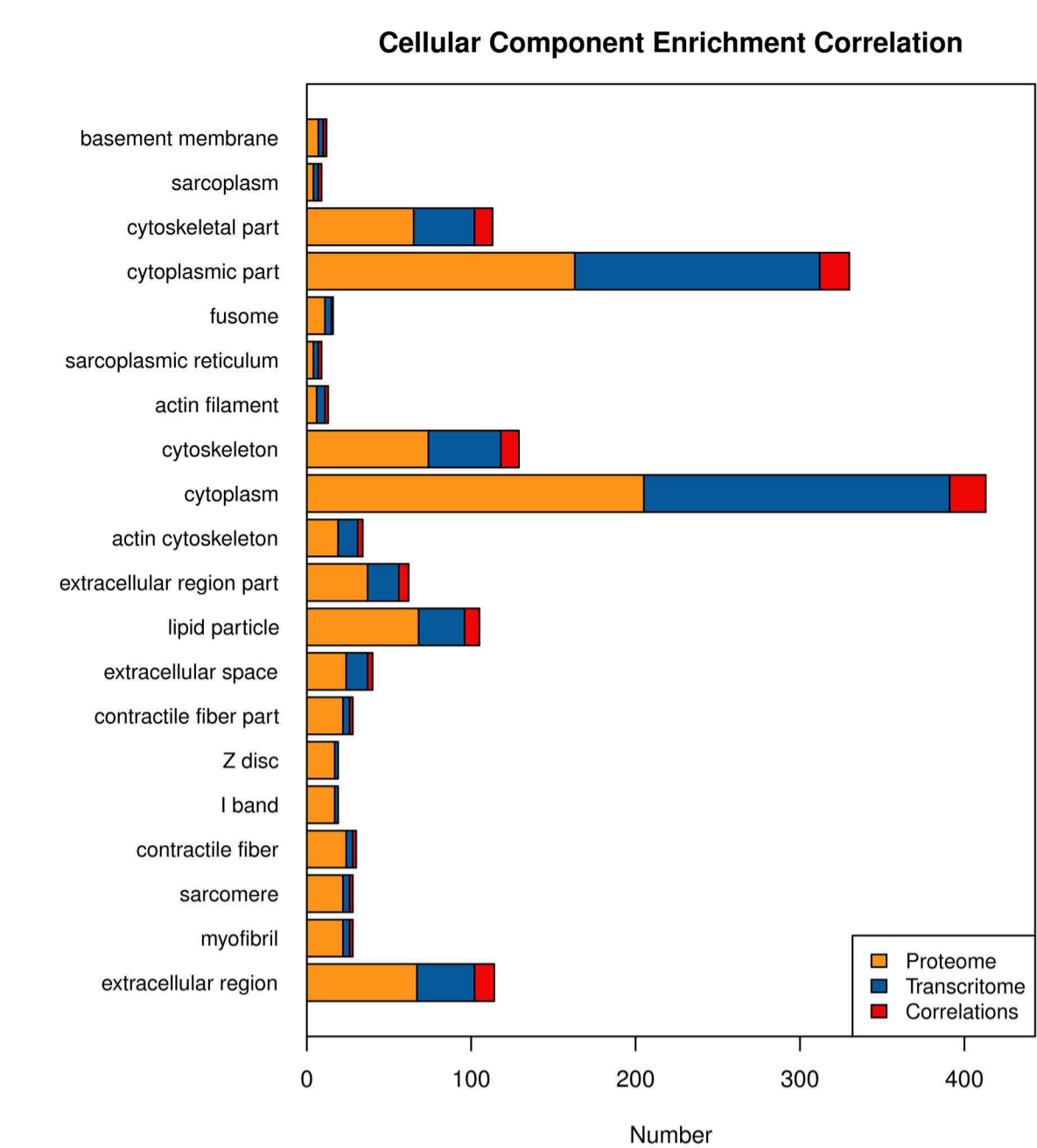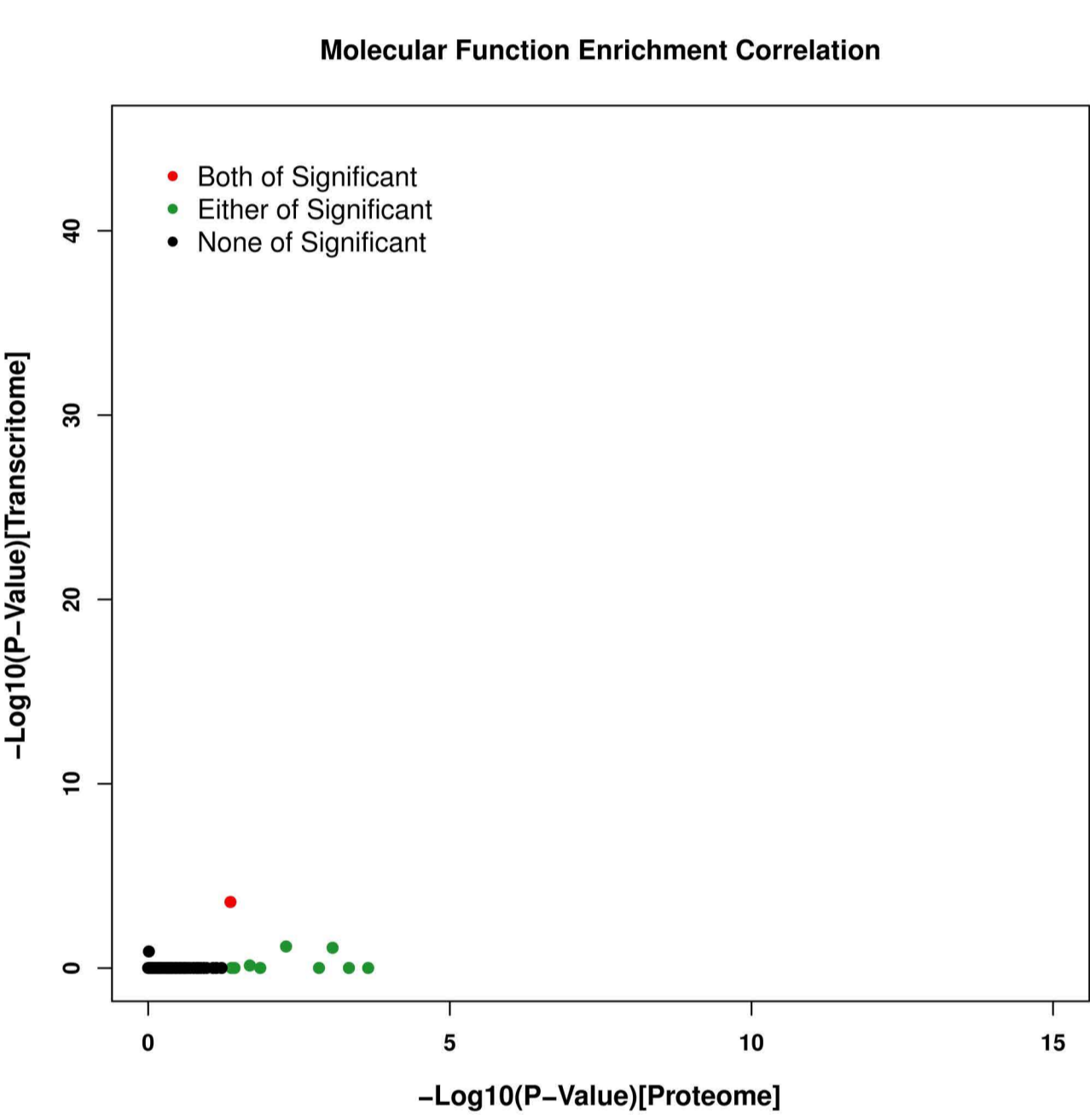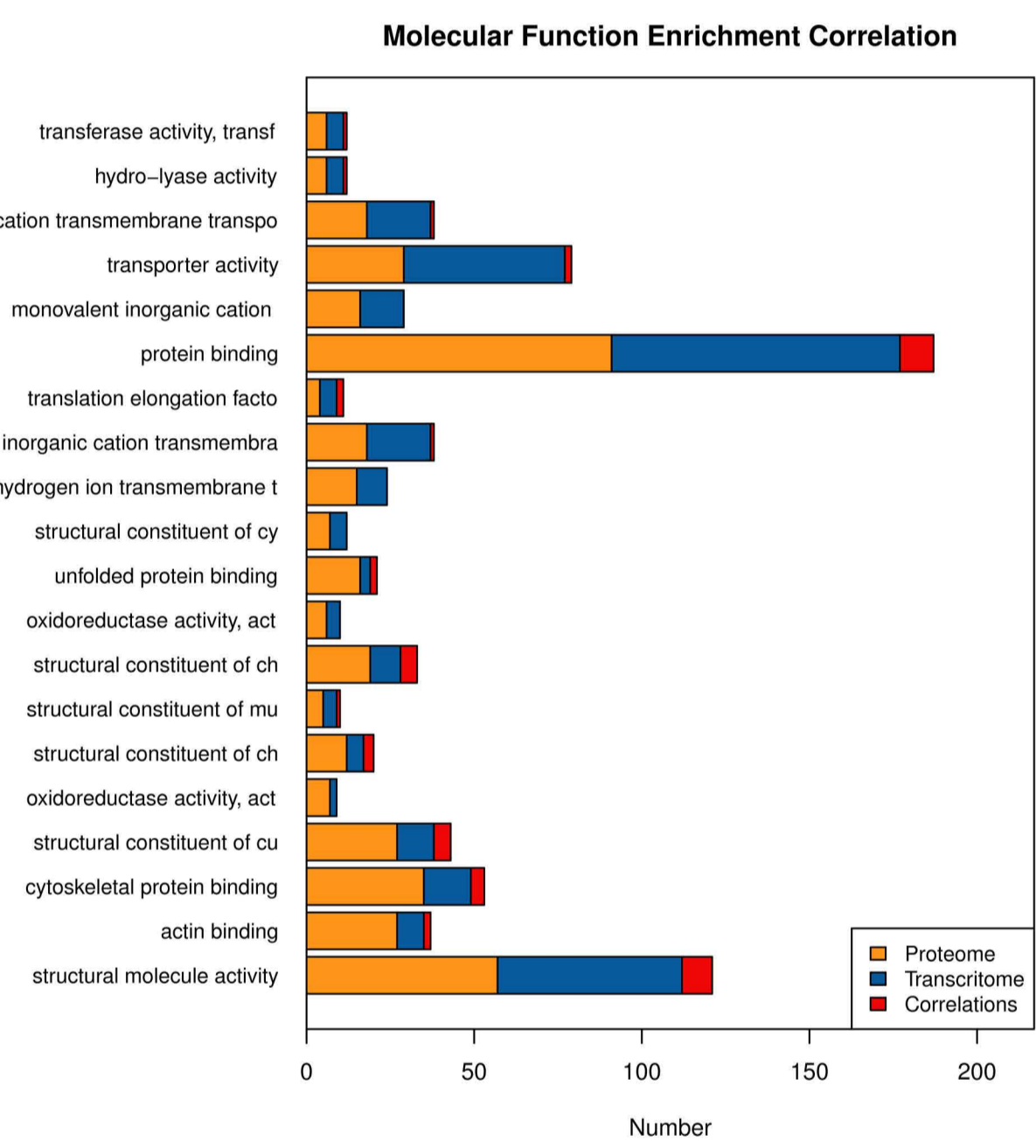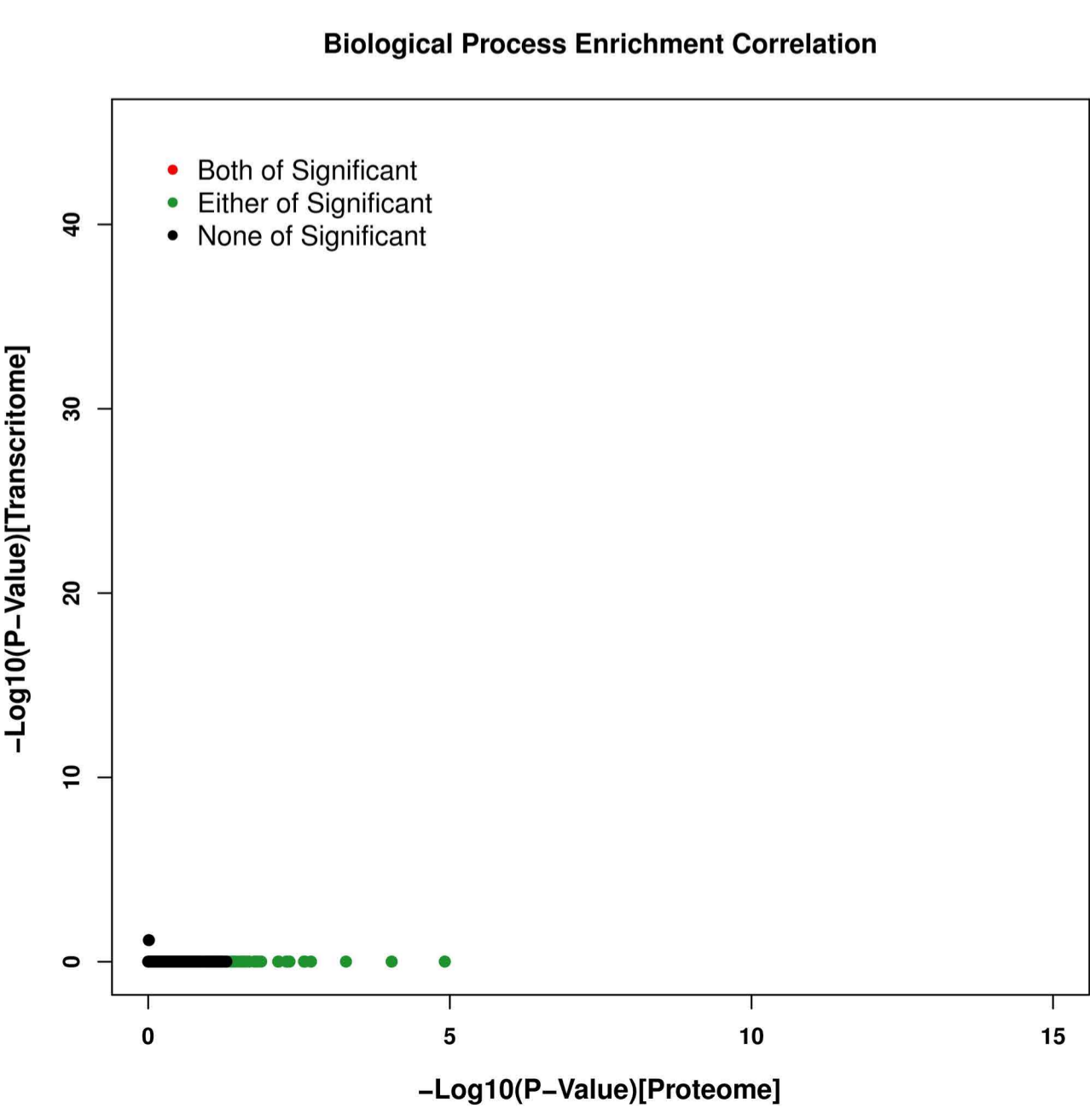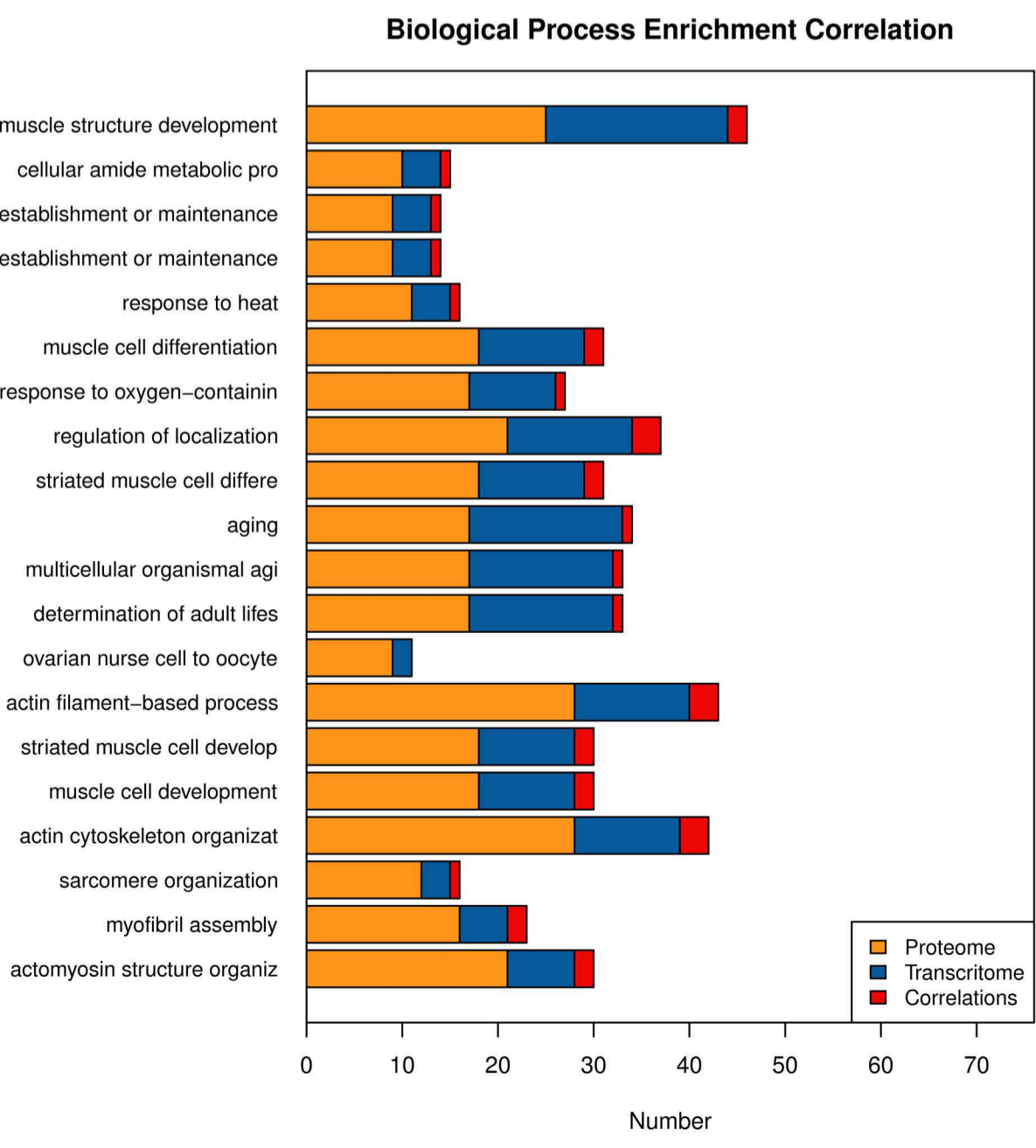

KEGG Enrichment Correlation

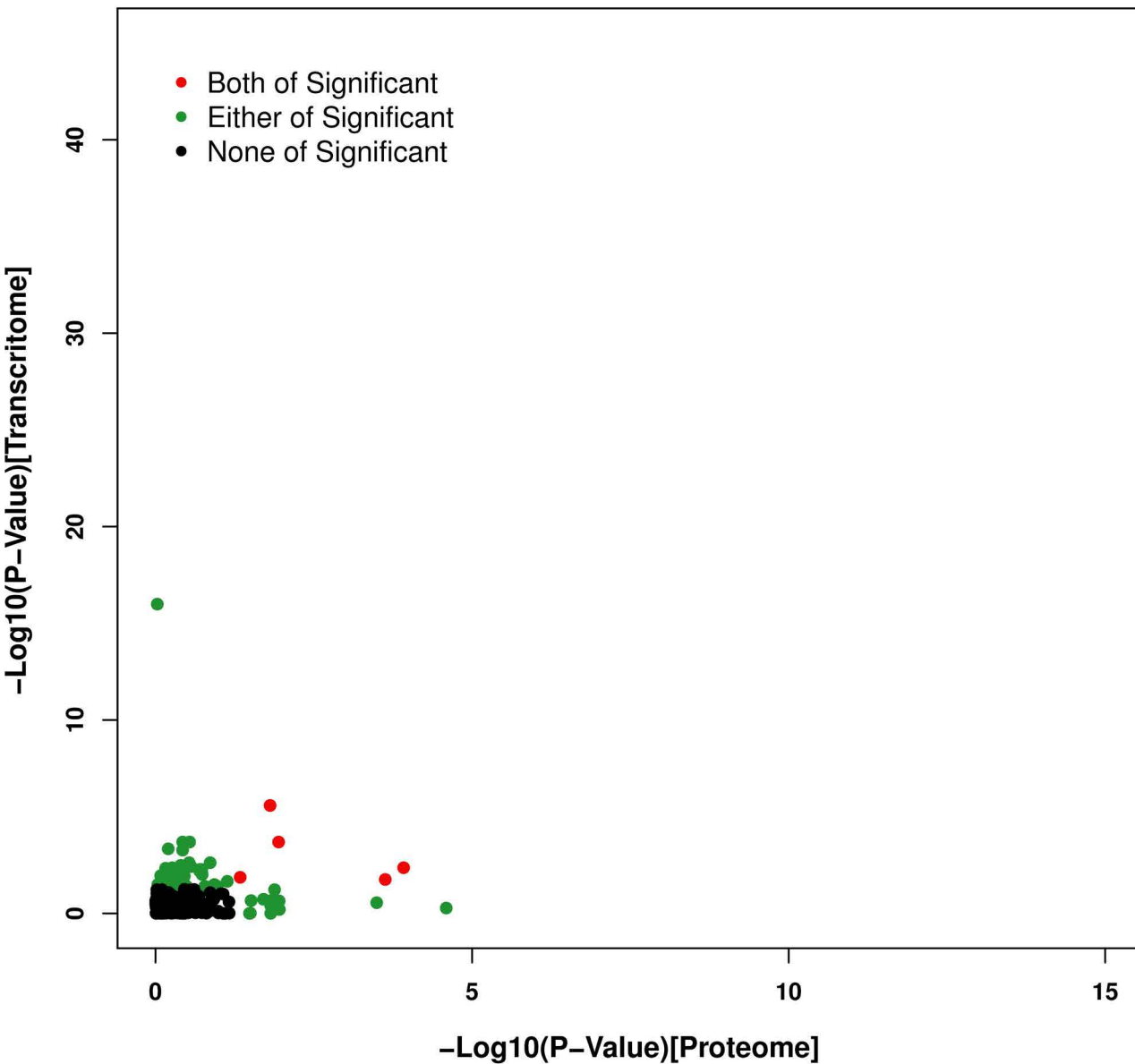

KEGG Enrichment Correlation

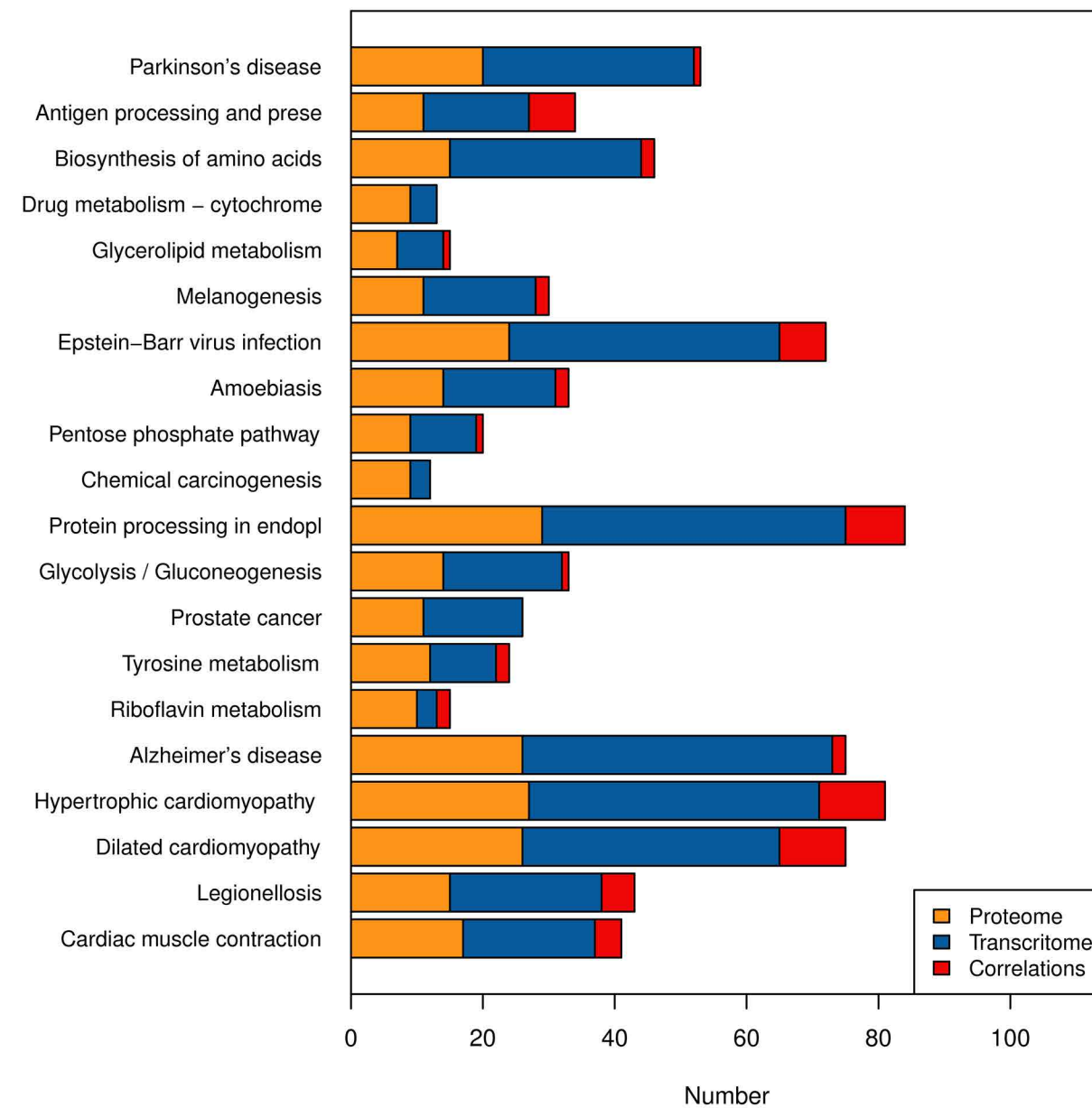

Supplement: Supplementary Data Sheet 1 — The GO and KEGG enrichment for the significant expressed proteins in transcriptome and proteome data. [file Data_Sheet_1.PDF]

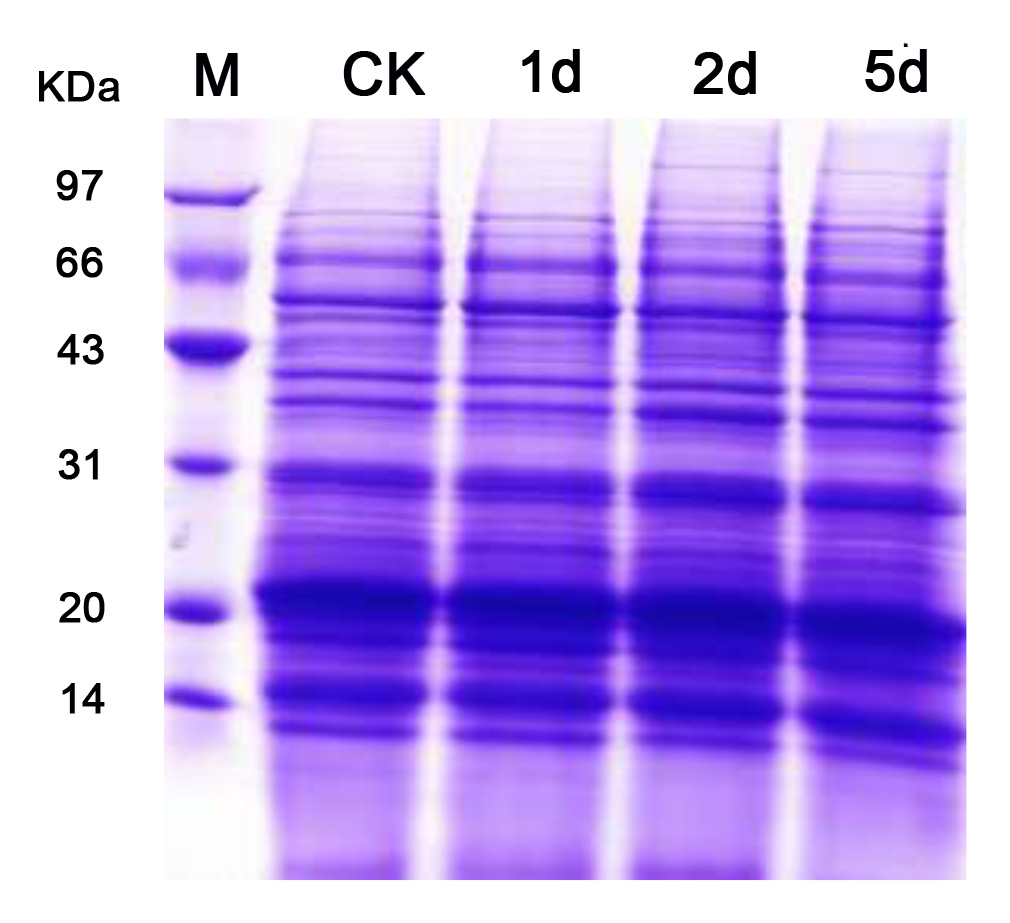

Supplement: Supplementary Image S1 — SDS-PAGE electrophoresis of the samples of Bactrocera minax pupae treated by carrier solution and 20-hydroxyecdysone at different time points. The samples were separated by 10% resolving gel in duplicate. [file Image_1.JPEG]
